# From Waste to Value: Recent Insights into Producing Vanillin from Lignin

**DOI:** 10.3390/molecules29020442

**Published:** 2024-01-16

**Authors:** Paola D’Arrigo, Letizia A. M. Rossato, Alberto Strini, Stefano Serra

**Affiliations:** 1 Department of Chemistry, Materials and Chemical Engineering “Giulio Natta”, Politecnico di Milano, p.zza Leonardo da Vinci 32, 20133 Milan, Italy; 2 Istituto di Scienze e Tecnologie Chimiche “Giulio Natta”, Consiglio Nazionale delle Ricerche (SCITEC-CNR), via Luigi Mancinelli 7, 20131 Milan, Italy; stefano.serra@cnr.it; 3 Istituto per le Tecnologie della Costruzione, Consiglio Nazionale delle Ricerche (ITC-CNR), via Lombardia 49, 20098 San Giuliano Milanese, Italy; alberto.strini@itc.cnr.it

**Keywords:** flavor, flavor authentication, lignin depolymerization, lignin valorization, photocatalysis, sustainable processes, vanillin

## Abstract

Vanillin, one of the most widely used and appreciated flavoring agents worldwide, is the main constituent of vanilla bean extract, obtained from the seed pods of various members belonging to the Orchidaceae family. Due to the great demand in the food confectionery industry, as well as in the perfume industry, medicine, and more, the majority of vanillin used today is produced synthetically, and only less than one percent of the world’s vanilla flavoring market comes directly from the traditional natural sources. The increasing global demand for vanillin requires alternative and overall sustainable new production methods, and the recovery from biobased polymers, like lignin, is an environmentally friendly alternative to chemical synthesis. The present review provides firstly an overview of the different types of vanillin, followed by a description of the main differences between natural and synthetic vanillin, their preparation, the market of interest, and the authentication issues and the related analytical techniques. Then, the review explores the real potentialities of lignin for vanillin production, presenting firstly the well-assessed classical methods and moving towards the most recent promising approaches through chemical, biotechnological and photocatalytic methodologies, together with the challenges and the principal issues associated with each technique.

## 1. Introduction

**Figure 1:**
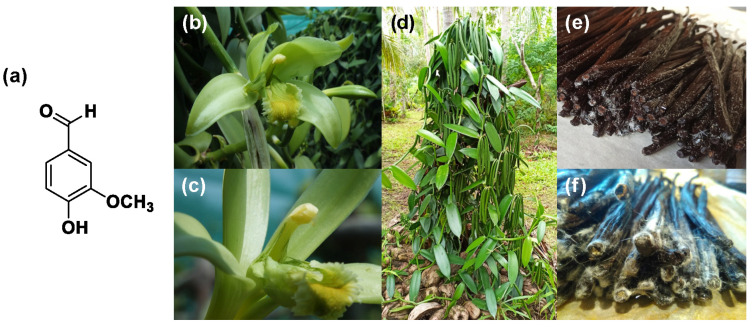
(**a**) Vanillin molecular structure; (**b**) *Vanilla Planifolia* Orchidacea after pollination; (**c**) vanilla Orchidacea after pollination flower details; (**d**) vanilla plant; (**e**) vanilla “frosted” pods; (**f**) vanilla crystallization details (original pictures kindly provided by Julien Pascal, New Caledonia).

Vanillin (4-hydroxy-3-methoxybenzaldehyde, CAS Number 121-33-5) is an aromatic aldehyde with different functional groups, such as carbonyl, ether and aromatic alcohol (see Figure 1a). It is a white solid, is soluble in water and constitutes the most important aroma component present in natural vanilla, providing its sweet and creamy odor.

The vanilla plant is originally from Mexico and is a tropical orchid of the Orchidaceae family, which includes more than 100 different species. As far as vanillin extraction is concerned, only three sources are relevant: *Vanilla planifolia*, *Vanilla pompona* and *Vanilla tahitensis* [1]. In particular, the first one is the most cultivated by the food industries because of its pod quality and yield, whereas the last one is the rarest and therefore the most expensive [2,3]. The cost of natural vanillin goes from a minimum of USD 1250/kg to 4400/kg.

Vanillin is the key constituent of natural vanilla flavoring whose fragrance profile is composed of more than 200 components, and it is one of the most diffused and expensive flavors around the world after saffron, with a widespread use not only in the food and beverage industry, but also in the pharmaceutical industry as masking agents, fragrances and cosmetic sectors [4]. Recently, the bioactive properties of vanillin such as neuroprotection and antioxidant, anti-inflammatory and anticarcinogenic activities have gained attention and increased its possible applications [5]. Moreover, vanillin has demonstrated great potential as a building block for polymer preparation and, in particular, for those which are composed of aromatic moieties [6,7]. In fact, nowadays, polymers based on renewable sources are mostly aliphatic ones, while polymers composed of aromatic compounds are usually petrol-based. For this reason, studies on the utilization of renewable aromatic materials such as vanillin derivatives are highly required in order to find an ecological solution to the present requirements of industry and civil society [7,8,9]. Vanillin can be inserted in polymer preparation as a monoaromatic monomer as well as part of a dimer [7,10]. For all these reasons, its global market was evaluated to be USD 627 million in 2022 [11]. Moreover, geographically, the Asia–Pacific region constitutes the largest market for vanillin, with a particularly high concentration in China and India. It is expected that, due to the increasing population of this area, this part of the globe will continue to dominate the market of vanillin.

From a historical point of view, vanilla appeared in Mexico during the Spanish conquest of the Aztec population. In particular, the Aztecs were the first documented population to use vanilla as a drink flavor. When, in around 1520, the Spanish arrived with the conquistador Cortez, the legend states that they tasted a new drink composed of chocolate favored with vanilla and they decided to import this amazing beverage to Spain. Then, vanilla became very popular all around Europe. In the 1800s, the vanilla constituents coming from the harvesting of vanilla beans were identified, and the dominant one was vanillin which was present in a concentration of over 1–2% (*w*/*w*) in the pod [3]. However, even if the growing of vanilla orchids and the extraction of vanillin is definitely time consuming and labor intensive, this method is still utilized because the obtained flavor is unique and natural, and so it can be directly used in the food and beverage sector. Approximately 1000 kg of vanilla pods have to be treated to recover 2 kg of vanillin, and its cost is also highly related to the availability of the pods. Moreover, vanilla cultivation is extremely laborious, and so farmers are discouraged from cultivating it on a large scale. For this reason, nowadays, the production of natural vanilla extract represents less than 1% of the total vanillin production and amounts to around 50 tons/year worldwide. Madagascar is presently the largest producer of natural vanillin. Furthermore, in addition to the high price of natural vanillin, the global demand for vanillin far exceeds the supply that can be obtained from natural sources. Therefore, there is a significant growing interest in developing cost-effective and sustainable methods for its production. In the early 20th century, chemists developed a method to prepare synthetic vanillin starting from fossil hydrocarbons such as eugenol [12]. Currently, vanillin is predominantly produced through chemical synthesis using petrochemicals, enabling the production of huge quantities of vanillin at a significantly lower cost (100 times less expensive than natural vanilla). However, this process raises significant environmental and sustainability concerns, and the use of the final product in the food and the pharmaceutical industry is subjected to severe restrictions [7]. Different biotechnological strategies have been recently developed in order to overcome these problems, mainly based on microbial fermentation, the use of enzymes and the exploitation of renewable raw materials.

In this framework, the use of lignin, an abundant renewable resource, as a feedstock for vanillin production is an attractive alternative. Lignin is in fact the second most abundant biopolymer on Earth, constituting 15–30% of the mass of plant cell walls. It is a complex aromatic polymer characterized by its heterogeneity and recalcitrance. The current industrial processes of paper and pulp production generate large amounts of lignin as a by-product, with most of it being burned as low-value fuel. The valorization of lignin into high-value chemicals like vanillin is nowadays gaining more interest because it can contribute to a circular bioeconomy and reduce our dependency on fossil resources [13].

Recently many questions about lignin structure and depolymerization have been deeply studied and are close to find important responses. Furthermore, different processes of vanillin production and purification are continuously being explored [14,15,16,17]. Furthermore, lignin-derived products play an important role in increasing modern society’s reliance on renewable-based chemicals, fuels, and materials and in reducing the carbon footprint of products and processes. In fact, lignin is a bio-based aromatic amorphous polymer that is considered the natural glue which provides structural integrity to plants. It represents about 15–40% (*w*/*w*) of a woody plant’s dry matter and 17–24% of herbaceous plants, and its use as a raw material does not compete with the food market, an aspect which is becoming ever more crucial nowadays because of the lack of food in the world compared to the needs of the population. Moreover, one very important aspect which has to be underlined in this discussion is the fact that lignin-derived vanillin can be considered a natural product in many contexts, so it can be used in all situations where natural products are required or preferred (i.e., food, cosmetics and nutraceutical formulations).

The high interest in vanillin production and its properties among the scientific community is clearly demonstrated by the publication of thorough reviews focused on different important aspects of vanillin science in the last few years [12]. The relevant pharmacological activities of vanillin have been described by Anand et al. in 2019 [18] and then in 2021 by Arya et al. who analyzed the therapeutic prospects of this compound [19]. Recently, Iannuzzi et al. published a deep overview on its beneficial effects for human health; in particular, they described the antioxidant activity of vanillin in addition to its anti-inflammatory, anti-mutagenic, anti-metastatic, and anti-depressant properties [20]. They reported also that vanillin exhibits peculiar neuroprotective effects on chronic neurodegenerative diseases and neuropathophysiological conditions. Regarding vanillin production, Banerjee et al. described, in 2019, the biotechnological achievements in this field [21], and Martau et al. [22] described, in 2021, a possible process to obtain bio-vanillin. Jang et al. have just published an overview on the vanillin biosynthesis technologies, describing the biotransformation of ferulic acid using native microbial strains as well as engineered microbes [23].

The present review aims to cover a topic not explicitly addressed in the existing literature, focusing on the production of vanillin obtained solely from the transformation of lignin, with particular reference to the most recent research. Special attention was given to the growing importance of low-impact technologies that exploit waste resources in a circular economy context. The different approaches will be analyzed starting from the most established one (currently in active production) moving to the most innovative, currently under preliminary laboratory study, discussing their advantages and disadvantages. Additionally, at an introductory level, the reader will find an overview of the different types of vanillin of commercial interest as well as a description of specialized analytical techniques for identifying the actual origin of commercial flavors, an increasingly important aspect in a highly competitive market environment.

## 2. Types of Vanillin

**Figure 2:**
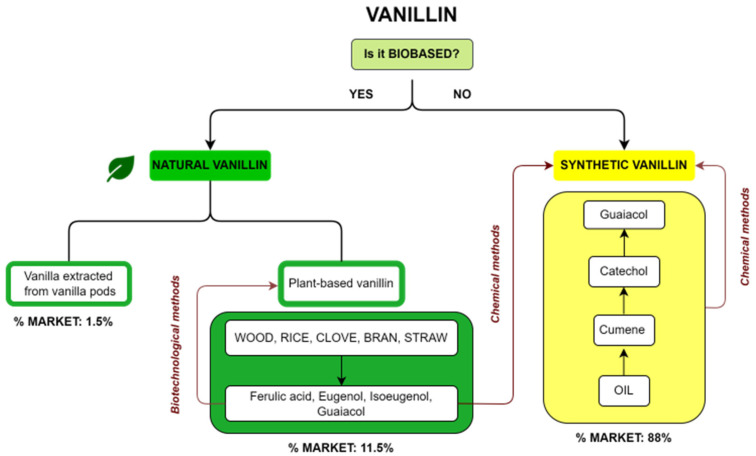
Diagram of the different types of vanillin, their sources and their respective market share.

Commercial vanillin can be categorized on the basis of its origin. A summary diagram of the main types of commercial vanillin including sources and market share is reported in Figure 2. The first question about vanillin production is whether it is a biobased product or not. In the first case, vanillin is defined as natural when the source is vanilla extract or vanilla pods or when it is a plant-based vanillin coming from the biotechnological treatment of compounds such as ferulic acid, eugenol, isoeugenol and guaiacol, obtained from natural fonts such as wood, rice, cloves or straw. On the other hand, if vanillin is not biobased, it is a synthetic vanillin obtained via chemical methods from products recovered from oil or from plant-based precursors. The market share for the different types of vanillin is reported in Figure 2. Synthetic vanillin accounts for 88% of the global market, whereas natural vanillin from pods accounts for only 1.5% and plant-based vanillin 11.5%.

### 2.1. Natural Vanillin

Current legislation aims to define what is considered “natural” based on the method of production. The criteria for naturalness may vary from state to state. In the past, flavors have been divided into three classes: natural flavors (extracted from natural sources or prepared from natural precursors using natural methods), nature-identical flavors (flavors produced via synthesis but chemically identical to natural ones), and artificial flavors (flavors produced via synthesis and not present in nature). Some countries, such as India and Brazil, still follow this classification. However, the natural flavoring industry is mainly influenced by the legislation of the United States and the European Union, which follow the international CODEX classification system. According to the Codex Guidelines for Flavoring CAC-GL 66/2008, natural flavoring substances are obtained through physical processes, such as distillation and solvent extraction, or enzymatic and microbiological processes from plant or animal material. These substances may be in their natural state or processed by traditional food preparation methods like drying, roasting, and fermentation. On the other hand, synthetic flavoring substances are formed through chemical synthesis. The European Union also classifies flavors into two categories: natural flavors and flavors. The reference legislation for this is Regulation (EC) No. 1334/2008 of the European Parliament and of the Council of 16 December 2008 on flavorings and certain food ingredients with flavoring properties for use in and on foods and amending Council Regulation (EEC) No. 1601/91, Regulations (EC) No. 2232/96 and (EC) No. 110/2008 and Directive 2000/13/EC. (Official Journal of European Union 2008, L 354/34). According to Article 3, paragraph c of this regulation, a “natural flavoring substance” is obtained through appropriate physical, enzymatic or microbiological procedures from material of vegetal, animal or microbiological origin. This means that natural flavoring substances are those that are typically present and identified in nature or are produced from natural precursors through natural methods.

Natural vanillin comprises both the vanillin extracted from the traditional source (e.g., *V. planifolia*) and the vanillin that is obtained starting from biological sources using selected processes that in several countries are considered, from a regulatory point of view, analogues to biological processes.

#### 2.1.1. Extraction from Vanilla Pods

**Figure 3:**
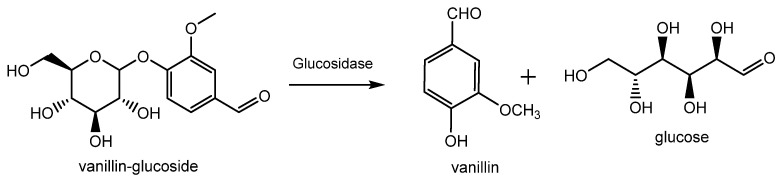
Production of natural vanillin from vanillin glucoside extracted from *Vanilla planifolia*.

Natural vanillin is recovered from the pod of a tropical orchid, especially the *Vanilla planifolia*, and is mostly produced in Indonesia, Madagascar, China and, to a lower extent, in Réunion, Guadeloupe, Turkey and Comores. Inside the green bean, vanillin is present as vanillin glucoside which is enzymatically hydrolyzed in vanillin and glucose during the curing process as shown in Figure 3 [12]. This process allows for the release of various vanilla flavor components with the ratio of vanillin accounting for 20 g/kg of vanilla beans. Thanks to this procedure, the resulting vanillin can be used without restriction for edible and pharmaceutical purposes and can be labelled as “natural vanilla flavor”. In this case, due to the high cost of this process, the price of natural vanillin can range from USD 1200/kg to more than USD 4000/kg.

#### 2.1.2. Plant-Based Vanillin

**Figure 4:**
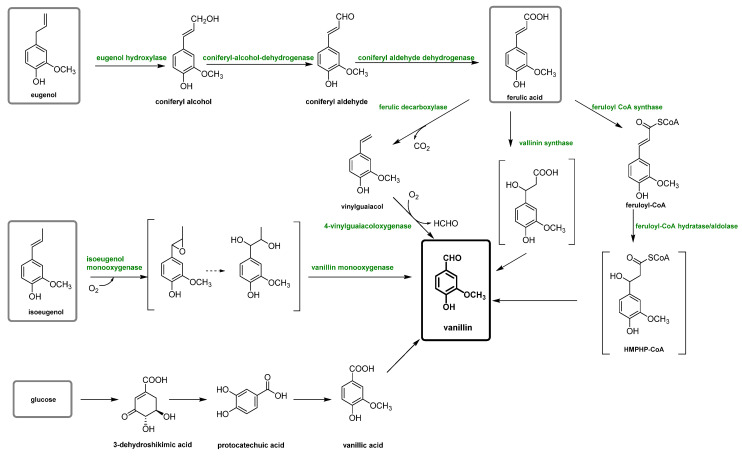
Scheme of vanillin production pathways through a biotechnological process.

Plant-based vanillin is the vanillin obtained biotechnologically using enzymatic or microbial transformations of non-oil-based precursors as carbon sources, such as ferulic acid, eugenol or glucose [23,24]. However, this type of vanillin can be considered natural or not, depending on the type of process utilized [12,22]. In fact, in the literature, only vanillin biotechnologically produced is considered natural and can satisfy both the US and the EU regulatory requirements. This vanillin is also frequently called “bio-vanillin”. The first example was reported in 1988, in which the precursor vanillic acid was obtained from a simple carbon source, glucose, using recombinant *E. coli* via the shikimic acid pathway [25]. However, recently, different biotechnological vanillin production technologies have been extensively investigated in order to obtain bio-vanillin. These techniques (summarized in Figure 4) include the transformation of eugenol, isoeugenol, ferulic acid and glucose with fermentation technology (solid-state fermentation), microorganisms bioengineering, enzymatic production, biosynthetic systems, bioconversion of agro-industrial wastes and production by microorganisms [26,27,28].

The price of vanillin obtained via the fermentation of ferulic acid is about USD 700/kg [29].

### 2.2. Synthetic Vanillin

**Figure 5:**
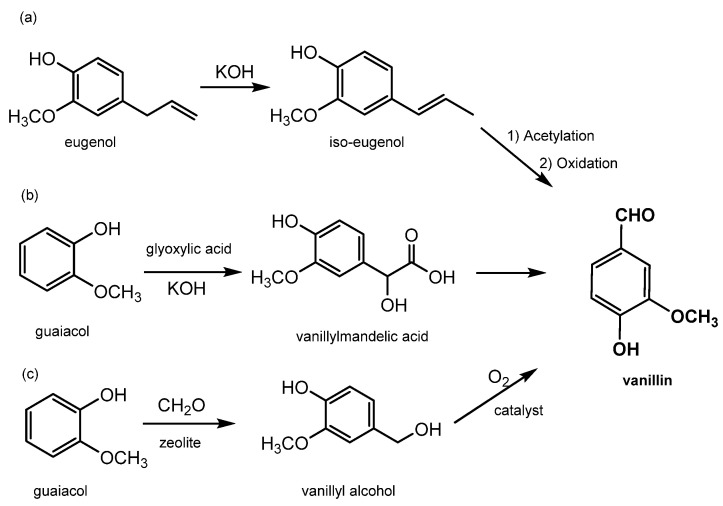
Main processes for chemical production of vanillin. (**a**) synthetic method to produce vanillin from eugenol by eugenol isomerization in isoeugenol and its oxidation; (**b**) vanillin production by Riedel process on guaiacol; (**c**) vanillin synthesis by guaiacol Solvay’s route.

Synthetic vanillin constitutes around 88% of the global vanillin demand, and its production starts from petrol-based intermediates, specifically eugenol and guaiacol; for this reason, it has to be labelled “synthetic or artificial vanilla flavor”. Additionally, the adjective “artificial” or “synthetic” makes it unappealing to consumers. Its price is relatively low, around 10–20 USD/kg [29], and it is sold mainly to ice-cream and chocolate manufacturers as well as in the form of fragrances to flavor companies. There are currently three main industrial processes to produce synthetic vanillin (reported in Figure 5). The first synthetic method produces vanillin from eugenol by exploiting the isomerization of eugenol to isoeugenol using KOH in diethylene glycol. Isoeugenol is then converted into an acetate for OH protection and then finally oxidized to form vanillin using nitrobenzene or potassium dichromate (as shown in Figure 5a) [2]. In the second method, which accounts for 85% of the total production, vanillin is prepared from guaiacol using the Riedel process which includes the condensation of glyoxylic acid into guaiacol to generate vanillylmandelic acid and then vanillin (see Figure 5b). This reaction is highly regio-selective towards the para position, thereby avoiding the formation of side products. The third method, known as Solvay’s route, involves the transformation of guaiacol, with a two-step reaction using sequentially HCHO and O_2_, into vanillyl alcohol at first and then into vanillin (see Figure 5c) [12].

### 2.3. Vanillin Authentication

As described before, vanilla pods, vanilla extract, natural vanillin and artificial vanillin have very different commercial values, ranging from USD 600/kg for Madagascar beans [30] (containing only about 2% by weight of vanillin) to a minimum of USD 10–22/kg for synthetic vanillin. This big price gap, as well as the increasing request for high value natural vanillin [30], has led to a booming growth of frauds, which relies on the replacement of natural vanillin by the cheaper and more easily available synthetic one.

Therefore, in the last forty years, a great number of scientific studies have faced the problem of determining the origin of this very important compound. These efforts have allowed us to acquire several new analytic tools for the authentication of the ‘natural’ rather than the “artificial” status of this flavor. From a chemical standpoint, there is no difference between vanillin deriving from vanilla plants and vanillin prepared through synthetic methods. Nevertheless, it is possible to detect a specific “chemical/isotopic vanillin fingerprint”, which depends both on the vanillin origin and on the chemical transformations that have been employed to produce a given vanillin sample.

#### 2.3.1. Chemical Fingerprint

Food or flavor preparations are considered authentic when they do not contain adulterants and their preparation method conforms to what is declared. This aspect is very relevant for any food and beverage containing vanillin or vanilla extracts. Indeed, vanilla extracts contain mainly vanillin and a few minor chemical components [31]. Although these compounds are present in very minute amounts, they contribute to the unique flavor of the natural raw material. Therefore, the chemical fingerprint of a given vanilla pod extract depends on many factors such as vanilla cultivar, geographic origin and method of production and can be employed as a reference standard for analytic purposes.

**Figure 6:**
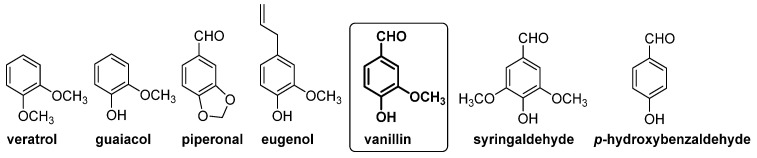
Compounds present in extract of vanilla pods (**left side**) and in barrel-aged alcoholic beverages (**right side**), which have been used as markers for vanillin authentication.

In this context, gas chromatography–vacuum ultraviolet spectroscopy (GC-VUV) [32] and capillary electrophoresis [33] have been used for the determination of natural and artificial flavoring compounds in natural extract samples. In these studies, guaiacol, veratrol, piperonal, eugenol, 4-hydroxybenzaldehyde, vanillic alcohol and vanillic acid were selected as chemical markers for the authentication of vanilla extract or vanilla aromatized foods (see Figure 6).

A similar approach has been proposed for the authentication of the origin of vanillin present in barrel-aged alcoholic beverages. It is generally recognized that this aldehyde gives an important contribution to the flavor of aged distillates [34] and, to a lesser extent, to aged wine [35]. In these products, vanillin originates from the degradation of the lignin present in barrels wood. During aging, lignin macromolecules release the monomers coniferyl, *p*-coumaryl and sinapyl alcohols. Coniferyl alcohol gives rise to coniferaldehyde, which is converted into vanillin and, in turn, is oxidized to form vanillic acid. Similarly, *p*-coumaryl and sinapyl alcohols generate the corresponding aldehydes, which are transformed into *p*-hydroxybenzaldehyde and syringaldehyde, respectively, which are further oxidized to their benzoic acid derivatives. Overall, the aging process affords a complex mixture of lignin-derived phenolic compounds, whose composition depends on several factors such as the kind of wood used, temperature storage, the alcohol content in the beverage and the duration of aging. Therefore, the analytic determination of these compounds in wines and distillates has turned out to be a useful tool for the assessment of their quality as well as to spot the fraudulent addition of synthetic vanillin [36].

#### 2.3.2. Isotopic Fingerprint

Obviously, the authentication of purified vanillin cannot be accomplished via an analysis of its compositional profile. In this case, the use of isotopic profiling has proven to be the most effective approach. Indeed, in an organic molecule, the distribution of stable isotopes is not statistical but depends on the synthetic/biosynthetic path of its formation.

Chemical and biochemical reactions proceed with a small but defined kinetic isotope effect, namely, the change in the reaction rate when one of the atoms in the reactants is replaced by one of its isotopes. Therefore, any chemical reaction can increase/decrease the content of a given isotope in the newly formed molecule, depending on the isotope effect of the specific transformation. Close to the most abundant isotopes ^12^C, ^1^H and ^16^O, vanillin contains the isotopes ^13^C, ^2^H and ^18^O, whose abundance and distribution in the molecular frame is strictly related to the synthetic method of its production.

**Figure 7:**
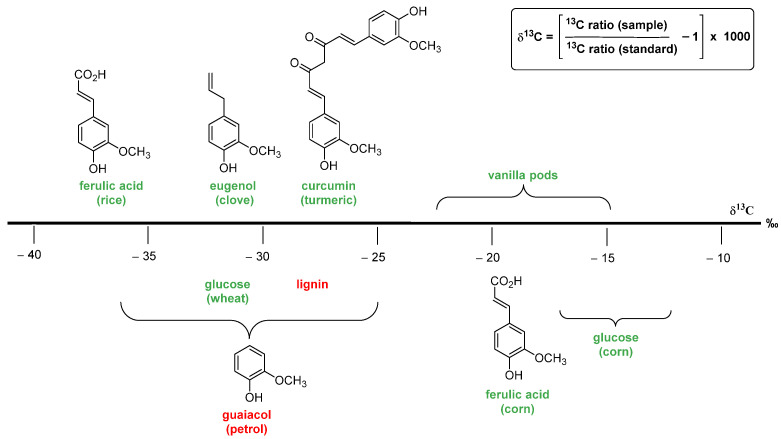
The fundamental precursors used for vanillin synthesis and the corresponding δ^13^C range of values measured for vanillin. The chemical precursors are reported in red; while the natural ones in green.

The authentication methods based on the carbon stable isotope ratio are widely used to assess the natural/artificial origin of vanillin. These analytic procedures are based on the evaluation of the ^13^C/^12^C ratio. For natural materials (plants, animals and minerals), the ratio value is approximately 0.0112, and only the last digit varies. Therefore, in order to have a more suitable index, ratio values are converted into δ^13^C value, calculated using the formula in Figure 7, and expressed in ‰ values. A more negative δ^13^C means more ^12^C, and a more positive δ^13^C means more ^13^C. Organic carbon contains less of the ^13^C, relative to the initial inorganic carbon from the atmosphere, because photosynthetic carbon fixation involves several fractionating reactions with kinetic isotope effects [37]. In plants, the δ^13^C values depend on the biochemical pathways of carbon fixation, namely, C3, C4 and CAM (crassulacean acid metabolism) photosynthesis mechanisms. In C3 vegetables, which are the most common type of plants, δ^13^C values range from −38‰ to −25‰, and in C4 plants (maize, sugar cane, millet, sorghum), the values range from −16‰ to −12‰, whereas in CAM plants, the values range from −20‰ to −10‰. The vanilla plant transforms carbon dioxide via the CAM pathway, and the reported δ^13^C values for vanillin extracted from vanilla pods range from −22.2‰ to −14.6‰ [38,39]. Since δ^13^C values of synthetic vanillin range from −36.2‰ to −24.9‰ (petrochemical) and from −28.7‰ to −26.5‰ (lignin) [40], the measurement of the δ^13^C value can be used to distinguish vanillin extracted from vanilla pods from synthetic vanillin. Unfortunately, the wide δ^13^C value range of synthetic vanillin overlaps those of natural vanillin deriving from the biotransformation of natural ferulic acid (from −37.9‰ to −35.4‰), eugenol (from −31.3‰ to −30.9‰) and curcumin (from −30.4‰ to −27.8‰) [41]. Moreover, it is worth mentioning that the δ^13^C value of a given sample can be fraudulently manipulated by adding synthetic ^13^C-depleted vanillin or by mixing samples with different ^13^C content.

Although isotope ratio mass spectrometry (IRMS) [40,42,43,44] still remains the most employed method for vanillin authentication, several new analytic approaches have been developed to overcome the above-mentioned issues. Taking into account that even ^2^H and ^18^O are incorporated in vanillin molecule with a measurable kinetic isotope effect, the combined measurement of the δ^13^C value with the δ^2^H value [39,45,46] or with δ^18^O values [47,48,49] have proven to be a useful tool in authentication. These approaches have turned out to be particularly effective when exploited in combination with NMR techniques. Indeed, the isotopic content of vanillin varies not only in total, but more importantly, as a function of the different atomic sites within the molecule [50]. This effect is the result of the specific synthetic/biological steps that have been involved in vanillin formation. Firstly, ^2^H [51,52] and recently ^13^C-NMR [53,54,55,56,57] have shown that it is possible to measure the isotope ratio of the specific atomic sites to obtain a complete isotopic fingerprint of the molecule. In this context, the SNIF-NMR (site-specific natural isotopic fractionation via nuclear magnetic resonance) methodology has been proposed to be the most valid NMR technique. SNIF-NMR and IRMS can be regarded as complementary analytic methods, and their combined use affords reliable results.

As a final point, it is worthy to describe the case of positional δ^18^O values. The sources of oxygen needed to build up every organic compound are CO_2_, atmospheric O_2_ and ground water. The δ^18^O values of these infinite reservoirs are very different from each other, ranging from +42.5‰ to +40.3‰ (CO_2_), from +23.8‰ to +23.5‰ (atmospheric O_2_) and from +2‰ to −10‰ (water) [58]. Moreover, three different oxygen atoms are placed in three different positions in the molecular framework of vanillin. Therefore, the positional δ^18^O values are strictly connected to the origin of the oxygen atoms that have been supplied in the synthesis/biosynthesis process.

**Figure 8:**
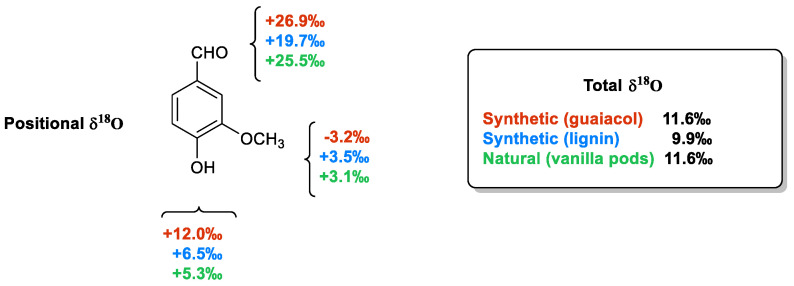
The positional δ^18^O values in three vanillin samples, two synthetic (from guaiacol and lignin) and one natural (from vanilla pods).

As shown in Figure 8 [47], the total δ^18^O values measured from vanillin samples of different origins are very similar to each other and do not allow for their authentication. On the contrary, the positional δ^18^O values are able to differentiate samples of synthetic origin from those extracted from vanilla plants or produced from lignin via chemical oxidation. The main difference between the samples derived from guaiacol and those possessing the aromatic moiety of natural origin is in the value of the oxygen atoms linked to the aromatic ring. Otherwise, the extractive materials from pods are distinguished from those derived from lignin based on the carbonyl oxygen δ^18^O values ranging from +26.2‰ to +25.5‰ in the natural material to +19.7‰ in the lignin-based sample.

Overall, all the above-described analytic methods have been successfully employed for vanillin authentication. In spite of this fact, the illustrated studies have underlined the difficulties in uncovering frauds related to the adulteration of this flavor. To date, the most successful approaches are those based on the combined use of at least two different analytic methods, which have higher chances to catch the most sophisticated frauds.

## 3. Vanillin Production from Lignin

**Figure 9:**
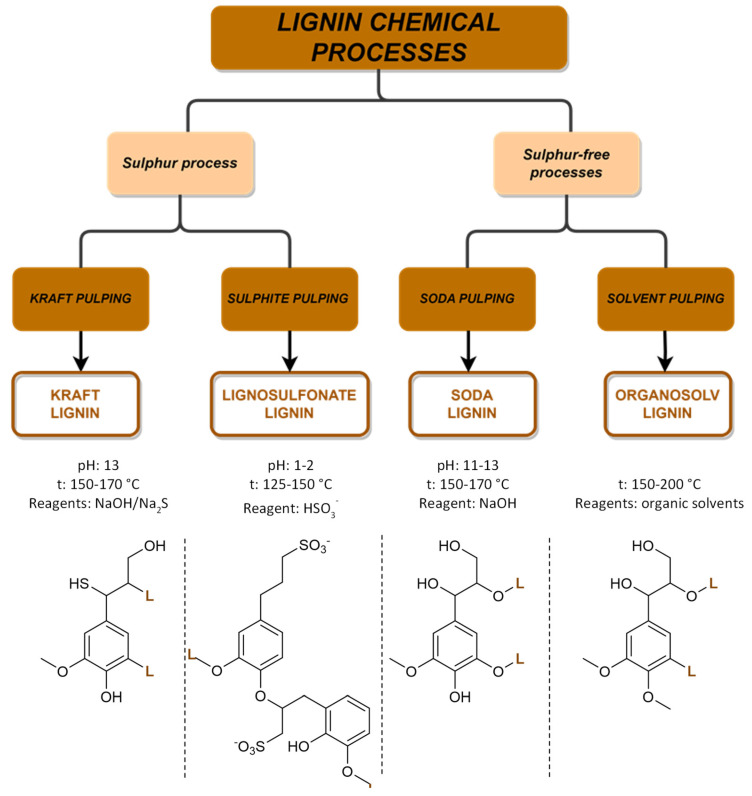
Overview of the four main chemical processes for lignin extraction and the structure of lignin.

Every year, over 50 million tons of lignin are extracted via wood pulping and other biorefinery industries, but only a small fraction of around 2% is utilized in various applications. However, with the growing adoption of extraction technologies and a shift towards biorefinery processes, lignin is increasingly being recognized as a ‘green’ feedstock for fuels, chemicals, and materials. Traditionally, lignin has been considered an industrial residue of pulp and paper factories, with most of the annual production being used as a low-cost fuel for power and heat generation. However, lignin-derived products can play an important role in increasing our reliance on renewable-based chemicals, fuels, and materials and in reducing the carbon footprint of products and processes. The structure of lignin is complex and varies depending on plant species, tissue type, and extraction methods. Research is ongoing to explore the potential of the different lignin sources with the challenge to identify the best starting materials along with a set-up of new technologies. The most common industrial available lignins and their extraction processes are summarized in Figure 9. The chemical processes are divided into two main groups related to the presence of sulfur in the extraction processes.

As the global economy continues to shift towards renewable feedstocks, there is a growing interest in developing new applications for lignin, which is driving commercial efforts. Moreover, from a circular economy perspective, the fractionation of lignocellulosic waste biomasses is also currently being highly investigated in order to recover and valorize the main components constituted by polysaccharides and lignin [59,60].

**Figure 10:**
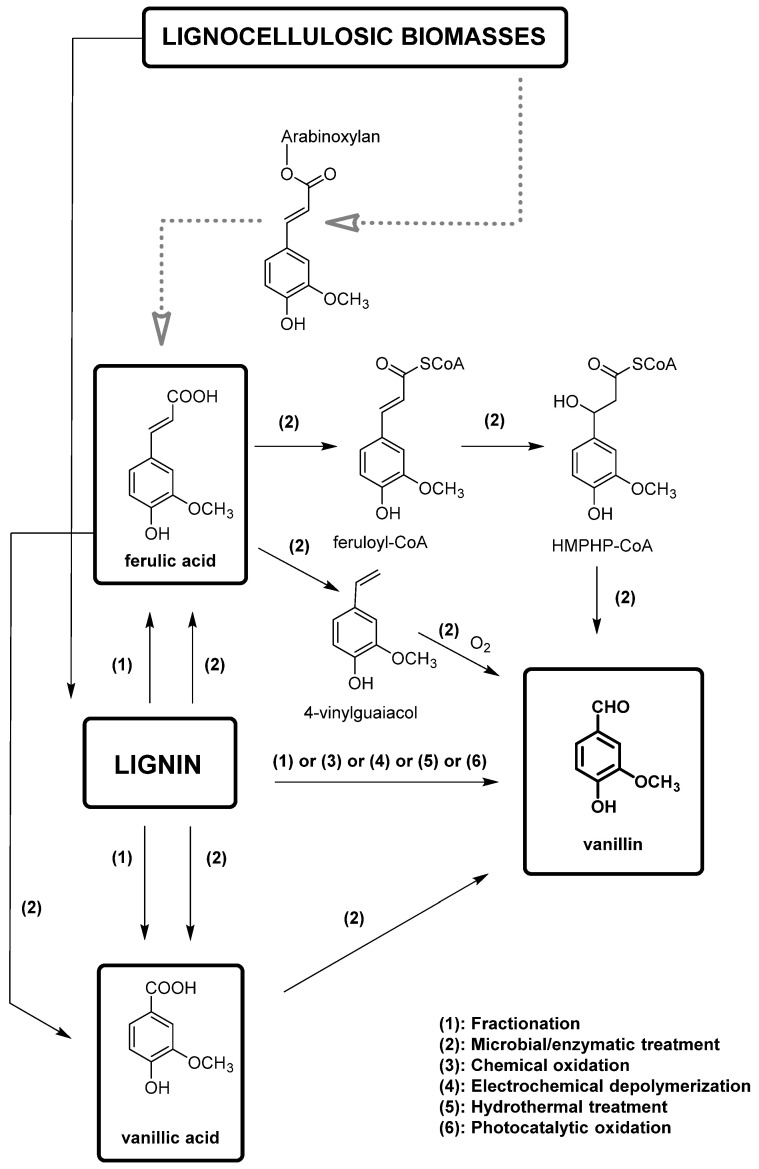
Overview of the production of vanillin from lignin derived from lignocellulosic biomasses (in gray, the production of ferulic acid from these biomasses is also reported).

In this framework, lignin-based vanillin is gaining importance also because oil-based vanillin is a non-renewable resource. However, the direct extraction of vanillin from pristine lignin, which could appear a simple obvious choice, results in a very low yield [61]. The process of producing vanillin from lignin involves breaking down the highly branched skeleton of lignin into smaller aromatic compounds, which can then be further converted into vanillin via chemical or biotechnological methods. Furthermore, 15% of the annual production of vanillin (around 20,000 t) is obtained from lignin. Chemical conversions typically require harsh reaction conditions and produce a wide range of by-products. The most important methodologies for the production of vanillin from lignin are summarized in Figure 10. Different methods have been developed including acid hydrolysis, oxidative depolymerization, and enzymatic hydrolysis and will be described here.

The unsatisfactory yield depends on the type of starting biomass and, in particular, on its content of β-O-4 linkages. In fact, Wang et al. analyzed the oxidation of five different lignins (Kraft lignin, alkali lignin, lignosulfonate and two enzymatic lignins) and several lignin model compounds to obtain vanillin, observing that the yield improvement was proportional to the content of β-O-4 linkages [62].

### 3.1. Lignin Oxidation

**Figure 11:**
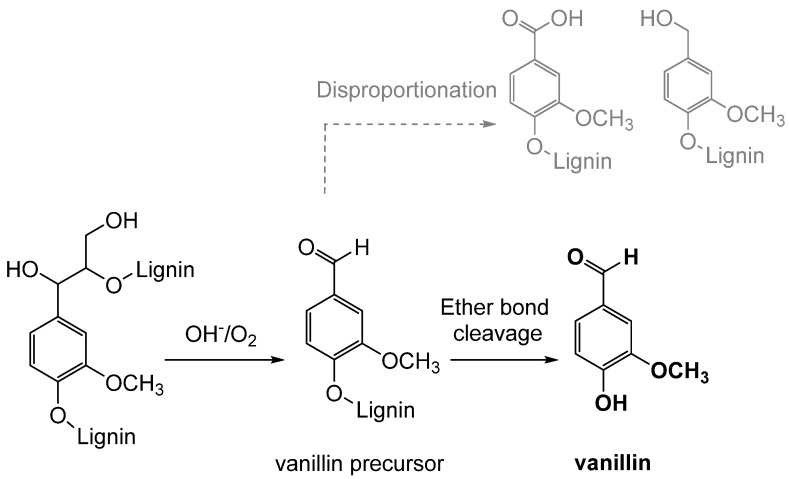
Simplified mechanism of lignin oxidation to form vanillin.

Oxidation is the classical process of converting lignin into vanillin through the use of oxidizing agents, such as oxygen, hydrogen peroxide, or ozone (see Figure 11). Industrially, the oxidative depolymerization of lignin is the most advanced and widely used technology for the production of aromatic compounds, including vanillin [63]. This process involves the oxidation of an aqueous solution of lignin, mainly lignosulfonate, which represents less than 10% of the total amount of lignins extracted worldwide [12]. Currently, only the Norwegian company Borregaard AS produces and sells vanillin obtained from lignosulfonate derived from the sulfite pulping of wood. This process is based on a simultaneous oxidation, using in particular molecular oxygen, and an alkaline hydrolysis of the lignin-rich fraction at high temperatures, high pH and in the presence of a transition-metal catalyst, obtaining a vanillin yield of 5–7% with respect to the starting material [64]. However, the reaction mechanism is not fully understood due to the complexity and the heterogeneity of lignin, and many parameters influence the production yields [65].

Researchers are exploring ways to valorize the main abundant Kraft lignin, which is produced in higher quantities than lignosulfonate, because it represents more than 90% of the global lignin production. In fact, this lignin is usually burnt for thermovalorization. A summary of the different classical methods for lignin oxidation is reported in different complete reviews and will not be rediscussed here [12,66,67].

Recent interesting studies have been conducted on lignin oxidation using always oxygen, which is environmentally friendly and not expensive, but with special attention to the tuning of all reaction parameters. In 2020, Gomes et al. reported the successful production of vanillin via alkaline wet depolymerization, performing at first an oxidation step on Kraft lignin (Indulin AT), followed by membrane ultrafiltration and chromatographic separation [68]. In this study, 40 L of a strong sodium hydroxide solution (80 g/L) containing 50 g/L of Indulin was oxidized in a packed bubble column reactor under 100% oxygen feed at 10 bar at 140 °C for 9.5 h. After the oxidation process, solid particles suspended in the solution were removed via membrane ultrafiltration. The solution was then subjected to membrane filtration with a 1000 Da cut-off, followed by a nanofiltration step with a 600–800 Da membrane at 60 bar. The chromatographic step was applied to the homogenized nanofiltrated permeate solution using a column packed with a non-polar SP700 resin. This phase allowed for the enrichment of the vanillin-containing solution by performing an alternated feed phase desorption using only deionized water without solvents. The study achieved an average recovery of 1.5 g/cycle, allowing for a vanillin recovery of 71% after 22 cycles with a final yield of 4.3% (*w*/*w* vanillin/lignin). In the same year, Khwanjaisakuna et al. recovered vanillin from Kraft lignin via its oxidation in a batch reactor using three different extraction methods: liquid–liquid extraction followed by distillation, two-stage liquid–liquid extraction and vacuum distillation [69]. The optimal reaction conditions were found to be 30 g/L of lignin, 110 °C and an oxygen partial pressure of 5 bar, resulting in a vanillin yield of 9.25% (*w*/*w*) after 67 min. Therefore, among the three methods, the liquid–liquid extraction one was found to be the most advantageous in terms of energy consumption and economic returns.

Lignin oxidation can also be conducted using particular catalysts in addition to the classic oxidizing agents. In 2018, Maeda et al. transformed lignin into vanillin in the presence of a non-classical catalyst, the tetrabutylammonium ion [70]. They oxidized lignin from Japanese cedar at 120 °C for 72 h with 1.25 mol/L Bu_4_N^+^ and 3.75 mol/L OH^−^. In these conditions, the vanillin yield was 7.2% (*w*/*w*), similar to the yield obtained via alkaline nitrobenzene oxidation, one of the most selective methods of transforming lignin into vanillin. This high yield indicated that the tetrabutylammonium ion was a specific catalyst for lignin degradation and vanillin formation. In 2020, Jeon et al. used a Mn–Cu mixed oxide catalyst and H_2_O_2_ as an oxidizing agent on Kraft lignin [71]. The alkaline wet oxidation was performed in a batch reactor using NaOH at 2 mol/L as an alkaline agent, 1 M H_2_O_2_ as an oxidizing agent and lignin at a concentration of 10 g/L. The oxidation was conducted at atmospheric pressure and at a temperature between 120 °C and 180 °C. They discovered that the optimal temperature for the highest vanillin yield was 150 °C, with a vanillin recovery of 6.8% (*w*/*w*), while the yield decreased at temperatures higher than 150 °C and particularly at 180 °C, due to the conversion of vanillin into the corresponding carboxylic acid. The Mn–Cu mixed oxide catalyst seemed to have better redox properties on lignin than classical ones. Zirbes et al. have just published a new two-step method for the oxidation of Kraft lignin with the green sodium peroxodicarbonate Na_2_C_2_O_6_ prepared in situ via the electrolysis of aqueous sodium carbonate and a subsequent thermal treatment which resulted in vanillin yields up to 6.2% and up to 92% referring to the maximum yield obtained from the quantification reaction using nitrobenzene [72]. Finally, Peng et al. proposed a co-solvent system to oxidize an alkaline lignin using a molybdenum-based solid as a catalyst and hydrogen peroxide as an oxidant. In particular, for lignin depolymerization, major yields were obtained when the water–THF system was used, increasing the total depolymerization products by 61%, with a total vanillin production of 6.5% [73].

Another important point that different authors have evaluated in the last few years is that, due to the highly heterogeneous nature of lignin, a possible strategy for its valorization is to first fractionate lignin into different homogeneous sharp molecular weight fractions, which can then be treated to recover or produce vanillin [61,74]. For example, Zhang et al. fractionated Kraft lignin using 1-propanol (1 g/60 mL) at 600 rpm for 4 h with a yield of 46% (*w*/*w*) and then oxidized the recovered lignin fraction using NaOH 2 M as an alkaline agent, CuSO_4_ 5H_2_O as a catalyst and H_2_O_2_ as an oxidative agent for 60 min at different temperatures. They found that, at 120 °C, the vanillin yield was above 9.4% (*w*/*w*), which was 40% higher than the vanillin obtained via the oxidation of technical Kraft lignin, and the yield increased until 10.9% at 140 °C [75].

Lignin oxidative depolymerization thus remains a promising method for the production of vanillin from lignin, but continued research and development of this technology are still necessary to face all the issues and to increase efficiency and yields.

### 3.2. Biotechnological Lignin Transformations

**Figure 12:**
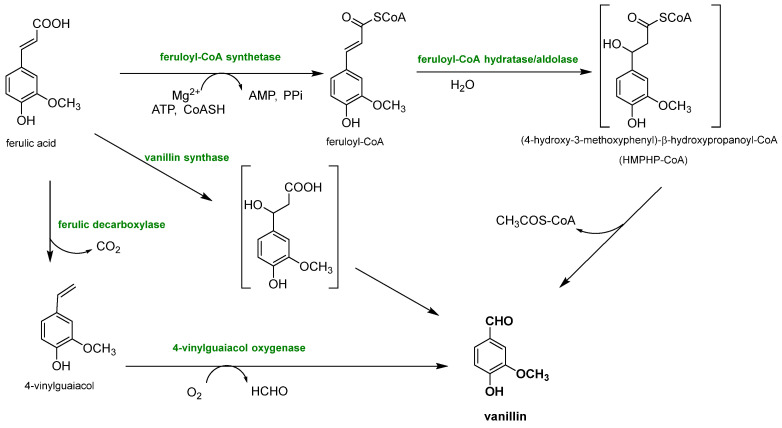
Different enzymatic reaction sequences for biovanillin production from ferulic acid recovered from lignocellulosic biomasses.

Biotechnological approaches have gained significant relevance for vanillin production from lignin, due to their sustainability and cost-effectiveness, because they permit the production of natural-identical vanillin which is in high demand due to the growing interest in natural and environmentally friendly compounds. This purpose is perfectly achieved by exploiting the biotechnological approaches, which involve the use of microorganisms, such as bacteria, fungi and yeasts, to transform lignin into the desired chemical products that perfectly fit this need. The main way to produce natural-identical vanillin through biotechnology consists of the microbial degradation of lignin to release ferulic acid which can be converted into vanillin (see Figure 12). In fact, ferulic acid (4-hydroxy-3-methoxycinnamic acid) is abundant in plant cell walls, and it is linked to lignin via ether or ester bonds. Its molecular structure is similar to that of vanillin, making the strategy of releasing ferulic acid from lignin and converting it into vanillin through biological valorization a valid option. Another great advantage is that, while the reduction of carboxylic acid to an aldehyde is difficult to obtain chemically, its microbial transformation is widely reported, less expensive and easier to manage.

Ferulic acid can be dissociated from lignin using feruloyl esterases (EC 3.1.1.73), a subclass of carboxylic ester hydrolases that catalyze the cleavage of ester bonds between ferulic acid and lignocellulose from common agricultural waste [76]. Feruloyl esterase activity was first discovered in 1987, and since then, it has been found in a wide range of microorganisms, such as bacteria (*Streptomyces olivochromogens* and *Fribrobacter succinogenes*) and fungi (*Penicillium* and *Aspergillus*) [77], and to date, over 30 microbial cinnamoyl esterases have been identified. It has been found that the distance between the phenolic ring and the ester bond, as well as the number and the position of the methoxyl and hydroxyl groups, influences the enzyme activity. Furthermore, maximum plant cell wall degradation is achieved when feruloyl esterase acts together with other hemicellulases, such as xylanases and pectinases. Once ferulic acid has been separated from the lignocellulosic biomass, it can be bio-converted into vanillin. Gram-negative bacteria (*Pseudomonas*), actinomycetes (*Streptomyces*) and gram-positive bacteria (*Rhodococcus*) are capable of producing vanillin from ferulic acid, but their yields are very low because vanillin is used as a source of carbon and energy by microorganisms and is rapidly converted into other products. To overcome these problems, microorganisms have been genetically modified, as reported by Di Gioia et al. who modified *Pseudomonas fluorescens* BF13 to promote vanillin accumulation by preventing its oxidation in vanillic acid [78]. They inactivated the gene encoding for vanillin dehydrogenase and amplified the feruloyl-CoA dehydrogenase gene. Under these conditions, vanillin production reached a yield of 1.28 g/L, the highest reported in the literature for *Pseudomonas* strains. Vanillin production can be also improved by using BF13 resting cells in successive conversion cycles and continuously recovering vanillin from the medium through a membrane system.

Since ferulic acid is so abundant in different agricultural wastes, Chattopadhyay et al. tried to extract it from wheat bran and then tried to consequently convert it into vanillin using *Streptomyces sannanensi* [79]. At first, ferulic acid was obtained using a ferulic acid esterase that cleaved the ester bonds present between the acid and the lignocellulosic biomass. Then, the acid was converted into vanillin (708 mg/L) through a CoA-dependent non-β-oxidative reaction sequence occurring via a CoA-dependent retro-aldol mechanism. In this case, unlike in the work of Di Gioia et al., vanillin was slowly converted into vanillic acid by the constant action of a vanillin dehydrogenase, which was not inhibited [11]. The main value of this study was that it not only produced vanillin using a biotechnological and sustainable pathway, but also utilized commonly available wheat bran, minimizing biovanillin costs and responding to the demand for environmentally friendly bioconversions [79].

Although lignin degradation in nature is carried out by fungi, in particular, basidiomycetes, they are not widely used for commercial purposes. Instead, bacteria are preferred, and the complexity of lignin requires the utilization of metabolic engineering and/or microbial consortia. In this way, different bacteria with different enzymatic activities can catalyze the cleavage of several bonds present in lignin and release the desired chemicals, avoiding the challenges associated with lignin heterogeneity [80]. Lignin-degrading bacteria can be found in nature in lignin-enriched environments, such as leaf litter, decomposing woods, compost soils, etc. Through bacteria-screening methods, some microorganisms belonging to phyla Proteobacteria, Actinobacteria and Firmicutes show a great ability to degrade lignin. These bacteria are able to depolymerize lignin using and combining the action of different enzymes, such as laccases, manganese peroxidases, Cyt P450, dioxygenase and others [81]. An example of using natural bacterial consortia has been recently reported by Baghel et al., who developed a new method to synthetize vanillin from Kraft lignin using five ligninolytic bacterial strains isolated from agricultural land [82]. Among them, only three showed ligninolytic activity at high concentrations of Kraft lignin. Lignin degradation and vanillin production were increased by combining these three strains rather than using them separately, and the maximum vanillin production (3.6% *w*/*w*) was obtained after 6 days of incubation at 35 °C and at pH 7.6.

Another example of utilizing natural bacteria consortia has been reported by Harshvardhan et al. [83]. They were able to produce vanillin and a few other products using bamboo chips (*B. tulda*) as a substrate for a natural bacterial consortium. They tested 14 natural bacteria consortia developed on bamboo chips using enrichment techniques, different media and different temperatures. Among them, only one bacteria consortia (H3) produced vanillin without any chemical pre-treatment. The consortium was composed of 28 strains identified by using the 16S rRNA sequencing method. Although vanillin was not the only product, it was the majority with a production of 0.9 ± 0.3 mg/mL vanillin, which can be compared to the vanillin yield obtained via the ferulic acid bio-conversion.

In addition to the bacterial consortium, a single bacterium can be used for vanillin production from lignin. Kaur et al. recently obtained vanillin from lignin extracted from two different biomasses (sugarcane biomass and coconut husk) using *Bacillus* sp. [84]. The authors extracted lignin from sugarcane biomass via acid hydrolysis (70:30 formic acid and acetic acid mixture) and from coconut husk via alkali hydrolysis (NaOH 1M) with a yield of 6% and 7%, respectively. Then, 1% of the extracted lignin was incubated in a mineral malt medium with 1% of *Bacillus* sp. at 37 °C for 48 h under shaking conditions, leading to a vanillin recovery of 0.7 g/L. This type of bioconversion is economically and environmentally advantageous and allows for the production of natural-identical vanillin.

### 3.3. Lignin Hydrothermal Liquefaction

Hydrothermal liquefaction (HTL) has been widely studied in recent years as a promising technology for biomass processing [85] and, more specifically, for lignin depolymerization [86]. HTL is based on the use of compressed water under subcritical (200–374 °C) and, less frequently, supercritical (>374 °C) conditions, at pressures typically in the 15–220 bar range. These conditions allow for the exploitation of the wide interval of dielectric constant exhibited by water, which drops from 80.3 to 17.5 in the 22 °C to 327 °C temperature range (at 200 bar), enabling the fine-tuning of its solvating capabilities with respect to a very wide range of organic compounds. To put this into perspective, the dielectric constants (at 20 °C) of methanol, ethanol, and acetone are 33.0, 25.3 and 21.0, respectively. Moreover, the water pK_W_ is also strongly dependent on physical conditions, decreasing from 14.0 (at 25 °C, ambient pressure) to 11.1 (at 300 °C, 250 bar) and thus resulting in a reaction ambient richer in hydroxide and hydronium ions. The combination of these properties allows for the establishment of a reaction medium that combines some characteristics of organic solvents with a certain degree of acid–base catalytic properties [87]. Further benefits of HTL comprise the relatively short reaction time, typically in the 1–60 min range [85], and its possible implementation as a continuous process [88]. Drawbacks include high installation and operating costs and the production of wastewater and solid residues (char) that must be valorized or disposed of [89]. Char formation is due to the recombination reactions of compounds in the aqueous phase [85] and can be addressed, to a certain extent, by the addition of a capping agent such as phenol [90].

HTL has been applied to lignin to produce bio-oils and/or phenolics [86]. The possibility to recover phenolic compounds indicates that, during HTL lignin depolymerization, hydrolysis is an important factor. For example, in bio-oils obtained from organosolv lignin, 80% of the products are monomeric and also dimeric phenolic compounds [86]. Lignin HTL is a promising technique, due to its ability to maintain functional groups with high selectivity using water as a solvent, which is not always possible with other processes. In particular, the utilization of supercritical water allows for the retrieval of bifunctional aromatic compounds [91]. The yield of the conversion of lignin into phenolic compounds also depends on factors not directly related to the HTL process itself, such as the source of lignin and the type of pre-treatment used. In fact, phenols, guaiacols and catechols are obtained from softwood lignin, whereas syringol is mainly produced from grass lignin.

The production of vanillin from lignin via HTL has typically been studied in the more general context of phenolic derivatives production. A selection of the most interesting approaches is presented here, with particular focus on the more recent literature.

Sebhat et al. [92] described a thorough study on the thermal liquefaction of Kraft lignin in several combinations of solvents (water and water/alcohol) and in the presence of various metal catalysts (platinum, palladium and ruthenium) with different support systems (alumina, zirconia and titania). The aim of the study was wide ranging, but here, only the results related to vanillin production will be reviewed. All measurements were carried out under inert atmosphere (argon), fractionating the products via acid precipitation and solvent extraction. In the experiments with pure water as the solvent (225 °C, 40 bar), they found a correlation of the process time (from 1 to 24 h) with the yields of both the organic phase and total monomer production. Not all monomer yields were, however, enhanced with longer process duration, and the best yield of vanillin was obtained after 1 h (0.29% *w*/*w* with respect to the original lignin). This is attributed to the probable degradation of several monomers in the process conditions (the most favored monomer in long time processes was guaiacol, with a 0.8% yield after 24 h). Temperature was also a critical parameter, with better results in monomer production obtained at 250 °C vs. 225 °C and a severe decrease in the yields at 275 °C. An increased production of insoluble fractions was, however, observed at temperatures above 225 °C. Experimental runs were then carried out in the presence of metallic platinum supported on different metal oxides (alumina, titania and zirconia). The effect of the catalyst and the different support was evident in the guaiacol yields, but only a modest enhancement was measured in the vanillin yields (0.33% with Pt/TiO_2_ vs. 0.30% without catalyst, 3 h, 225 °C). Slightly better results were found in a successive series with different metal catalysts, all loaded over ZrO_2_, where the authors found enhanced vanillin yields for Ru/ZrO_2_ (0.36%), Pd/ZrO_2_ (0.37%) and, interestingly, also in presence of ZrO_2_ alone (0.37%). The catalytic effect of ZrO_2_ in hydrothermal lignin liquefaction was also indicated in [90], but supplementary research is needed on this topic. Further experiments were carried out using water/alcohol mixtures (with either methanol, ethanol or isopropanol), obtaining remarkable increments in both the overall yield of organic fraction and the total monomer production. These conditions appeared however detrimental to the specific production of vanillin, which demonstrated a severe yield decrease in all runs with organic co-solvent.

Jia et al. [93] studied the catalytic hydrothermal liquefaction of lignin with metal catalysts (Ni, Fe and Co) supported over carbon nanotubes (CNT). The conversion was carried out in three different solvents (water, methanol and ethanol) at three different temperatures (260, 280 and 300 °C) for 30 min at 100–180 bar. The authors determined the yields of the obtained oil fraction. The best yields were found using ethanol at 280 °C. In these conditions, the Co/CNT and Ni/CNT catalytic systems gave the best yield, followed by Fe/CNT, CNT alone and the non-catalyzed process. A semiquantitative GC-MS analysis of the content of monomers in the oil fraction was carried out for all experiments conducted with ethanol at 280 °C, in which vanillin was found to be the main compound. Quantitative determination of the actual vanillin yield, however, was not performed.

More recently, Cui et al. proposed an HTL method based on Ni-impregnated ZrO_2_, CeO_2_ and MgO catalysts to depolymerize a pinecone lignin into a bio-oil. The major yields of bio-oil (65.7% *w*/*w*) were obtained when 3% (*w*/*w*) of Mo was used together with 5% of the Ni/CeO_2_ catalyst. They tested also the effect of several solvents and found out that the methanol/water (1:1) mixture allowed them to obtain 77.8% of bio oil at 280 °C/30 reaction conditions. Of this recovered bio-oil, GC-MS analysis confirmed that 67.4% was vanillin [94].

The use of a combination of supercritical CO_2_ (scCO_2_) and subcritical water (sbcrH_2_O) as the solvent for lignin hydrothermal liquefaction was studied by Numal-Al-Mobin et al. [95]. The process was carried out at 220 bar at different temperatures in the 250–350 °C range for a 10 min nominal residence time using alkali (Kraft) lignin as the starting material. Several water:CO_2_ proportions were tested between 1:5 and 2:1 ratios. After the thermal treatment, the water medium was extracted with dichloromethane and analyzed via GC-MS with a relative determination of the various monomer content in each run. In these conditions, the authors found evidence for the selective production of specific monomers, including vanillin, guaiacol, *p*-propylguaiacol and *p*-ethylguaiacol. Particularly, in the run at low temperature (250 °C) with the highest CO_2_ content, vanillin was found to be the main reaction product (33% relative abundance between the other compounds found in the GC-MS run). Higher temperatures demonstrated a selectivity shift toward guaiacol. A decrease in the CO_2_ fraction also caused a decrease in the vanillin relative yield. The temperature and water:CO_2_ ratio was thus a powerful combination for the selectivity control in this approach. The advantages of using the scCO_2_/sbcrH_2_O combination were attributed to its inclination to form an acidic medium with homogeneous catalytic properties capable of an efficient cleavage of the β-O-4 bond in the lignin backbone. Moreover, the supercritical CO_2_ physical properties promoted an improved penetration into small lignin pores, enhancing the completeness of the reaction. Interestingly, the use of the scCO_2_/sbcrH_2_O combination appeared to suppress char formation up to 300 °C, superseding the need for capping agents. Also, a series of runs carried out at different residence times (1, 10 and 20 min, respectively) indicated that a quick treatment could be beneficial for the relative vanillin yield, while longer treatments favored the production of guaiacol. The lack of global yield data prevented, however, a direct correlation of these results with other approaches. In a related work, Rajappagowda et al. [96] studied the heterogeneous catalytic liquefaction of lignin in sub/supercritical medium (water with supercritical CO_2_ or N_2_). Here, the processes were carried out in the presence of a heterogeneous catalyst, either NiO or ceria-doped scandia-stabilized zirconia (CeScSZ), in a temperature range of 200–400 °C with water and CO_2_ or N_2_ maintained at 220 bar. Each run lasted for 10 min. The organic fraction was separated using dichloromethane and analyzed using GC-MS, always with relative quantification of each peak with the ratio between peak area and cumulative area of total ion current in each chromatographic run. Among the two catalysts, NiO was found to be the best performer in terms of both total phenolic yield and relative vanillin yield. Particularly, the run at 200 °C with NiO and scCO_2_ demonstrated the best vanillin selectivity. As has been previously found, higher process temperatures caused a decrease in the relative vanillin yield. In nearly all the runs, the substitution of CO_2_ with nitrogen decreased both the total phenolic and the relative vanillin yields, confirming the beneficial role of supercritical CO_2_ demonstrated in the previous study.

The results of the two previous works [95,96] constitute the basis for the analysis carried out by Isola et al. [97] on the sustainability of vanillin production from lignin using the described hydrothermal liquefaction processes. In this study, a comparative process involving simulation and the life cycle assessment (LCA) was carried out considering several experimental parameters, including temperature, reaction time, process atmosphere, lignin loading, catalyst type and vanillin yield. The latter parameter was calculated from the extracted oil weight, interpolating missing values. The analysis emphasized the value of shorter reaction times (because, in this case, the vanillin yield gain was dominated by the increased energy consumption) and the use of scCO_2_ at a relatively low temperature (200 °C). Interestingly, despite the obvious environmental drawbacks correlated with the use of metal-based catalysts, the use of NiO, in combination with scCO_2_, resulted in the lowest overall environmental impact. Moreover, the selection of a more environmentally friendly solvent for the final extraction step (e.g., ethyl acetate instead of dichloromethane) could further improve the sustainability of the processes. The best overall performance, among the processes considered in this study, was shown by the NiO/scCO_2_-based process carried out at 200 °C with a residence time of 10 min. Undoubtedly, the validity of any model is always limited by the validity of the data and assumptions on which it is based, yet this kind of analysis could be very useful in putting into context, both environmentally and economically, the different contributions of all the factors involved. This could be particularly important in the preliminary assessment of nascent technologies, which, by definition, cannot rely on prior historical experience. Further studies in this field are therefore certainly welcome, both on the modelling side and the experimental data production side.

The prospects in the application of lignin liquefaction for the production of vanillin or other high value-added intermediates thus appear quite interesting, particularly considering the favorable environmental impact due to the use of water as the main process solvent. Moreover, most studies indicate a favored vanillin production using relatively low reaction temperatures, which further improves the process sustainability. Major limiting factors on the environmental and economic scalability of this approach include, however, the high energy use (due to high process temperatures) and the use of organic solvents in final product extraction. Future developments therefore should comprise, among others, the study of improved catalysts that can keep process temperatures and times down [85] and the optimization of final product separation, either by identifying improved solvents or by using alternative techniques. The implementation of a continuous process is also particularly interesting, but some challenges must be addressed to put into practice this technology [89].

### 3.4. Lignin Electrochemical Depolymerization

Electrochemical depolymerization is considered one of the most promising techniques for producing aromatic fine chemicals via a lignin cleavage. In fact, this process, which is an anodic degradation, is particularly environmentally friendly because it does not need intensive energy-consuming pre-treatments, such as high temperature and pressure, and can use renewable energy as a power source [98]. Moreover, this method overcomes the major issues associated with the production of bio-based vanillin linked to the use of toxic reagents and the presence of waste by-products that could contaminate the desired final vanillin. However, this technique is not widely exploited due to the low yield it produces which is conditioned by the nature of the electrode responsible for mass transport and flow distribution and by the electrochemically active area. Active nickel-based electrodes were tested in 2016 by Stiefel et al. on Kraft lignin [98]. They experimented with these electrodes at room temperature, ambient pressure and using a current of 8 A, obtaining a lignin degradation of 81% in 2 h, 87% in 3 h and 96% in 11 h [98]. The reduction of lignin molecular weight to 220 Da was achieved, but they recovered, using a membrane module containing polymeric tight ultrafiltration membranes in a constant-flow cross-flow mode, vanillin, acetovanillone and different carboxylic acids with an individual yield of these products lower than 0.5%. The authors reported that this method was still in development and the yields could be improved in order to use the technique on an industrial scale.

In 2020, Zirbes et al. investigated a new electrochemical method to perform the selective electrodegradation of Kraft lignin to obtain vanillin and acetovanillone [99]. They used a simple undivided high temperature electrolysis cell, where lignin was dissolved in NaOH 3 M at 160 °C. The current of electrolysis was constant at 10 mA/cm^2^ (60 mA, applied voltage range 2–3 V) using planar Ni-charged electrodes. Then, the mixture was cooled at room temperature and acidified, and the products (vanillin and acetovanillone) were extracted with ethyl acetate. The charge/mass ratio of 2.7 C/mg of lignin and the selected temperature allowed for an increase in the electrolytic conversion, and the vanillin yield reached a value of 3.0% (*w*/*w*) which corresponds to 67% efficiency compared to the common nitrobenzene oxidation. This study demonstrated that this “green” technique based on sustainable electricity could replace classical oxidation, avoiding the use of toxic and carcinogenic reactants, and could also be applied to different types of lignin, with good yields in biobased vanillin in the range of 2.2–4.2%.

In the same period, Yan et al. investigated the electrochemical depolymerization on a different substrate constituted by organosolv lignin isolated from three different biomasses (sweetgum, aspen and loblolly pine) [100]. They used Ni electrodes because of their low competitive cost and their possibility to obtain high conversion yields. In particular, ethanol organosolv lignin (EOL) was obtained via precipitation from an ethanol organosolv-pretreated hydrolysate of each lignocellulosic biomass. Then, EOL was subjected to electro-oxidation in KOH 1 M using three different electrodes: a Ni-charged one as the working electrode, a Hg/HgO one as the reference electrode and a Pt wire as the counter electrode. The substrates were initially subjected to cyclic voltammetry and then to long-term electrolysis at a constant potential. In the end, after acidification, the products (vanillin and syringaldehyde) were extracted with chloroform with a total maximum yield of 17.5% for the electrolysis of EOL recovered from sweetgum.

In 2021, Di Fidio et al. conducted an electrochemical depolymerization of soda lignin Protobind 1000 [101]. This study provided insights into the optimal conditions and parameters required for processing this particular lignin. The researchers used a double-walled divided electrochemical glass cell, with one cell containing the catholyte and the other the anolyte. An ion exchange membrane was used to separate the two cells. The selected “three electrodes configuration” consisted of a Ni counter electrode, a Ag/AgCl reference electrode and a Ni/NiOOH working electrode. Argon gas was purged in the electrochemical cell prior to and during the electrochemical measurements in order to completely remove oxygen, avoiding the involvement of the atmospheric oxygen in the investigated reaction. The best conditions for lignin depolymerization were found to be pH 14, 20 g/L of the substrate and a voltage of 0.4 V. Under these conditions, it was possible to recover vanillin (0.12% *w*/*w* loaded lignin) and also other important monomers, such as sinapic acid (0.32%), vanillic acid (0.12%) and acetovanillone (0.15%). The sum of the concentrations of the aromatics thus resulted in a production of around 1.2 kg of total aromatics from 100 kg of Protobind 1000 lignin. This yield value of 1.23% was also in line with the literature.

An interesting possibility is to combine hydrogen production and lignin depolymerization. In the ordinary electrocatalytic water splitting for hydrogen production, the anodic product stream is oxygen. By replacing the oxygen evolution reaction (OER) with a less energy-intensive process such as biomass depolymerization, it is possible to implement a process with less energy requirement and with an anodic product stream of greater value than oxygen (e.g., phenolic in the case of lignin depolymerization). Ghahremani et al. [102] studied the simultaneous hydrogen production and lignin depolymerization using NiSn electrodes and lignin dissolved in 1 M NaOH solution. The authors studied different Ni/Sn ratios and cell potentials and found the highest vanillin production rate (about 300 mg/min of lignin at 10 g/L lignin concentration) using the NiSn_20%_ at 1.4 V. Unfortunately, no yield data were provided for a complete batch process example. Higher cell voltages result in a lower lignin production rate because of the activation of competitive anodic OER.

### 3.5. Lignin Photocatalytic Depolymerization

**Figure 13:**
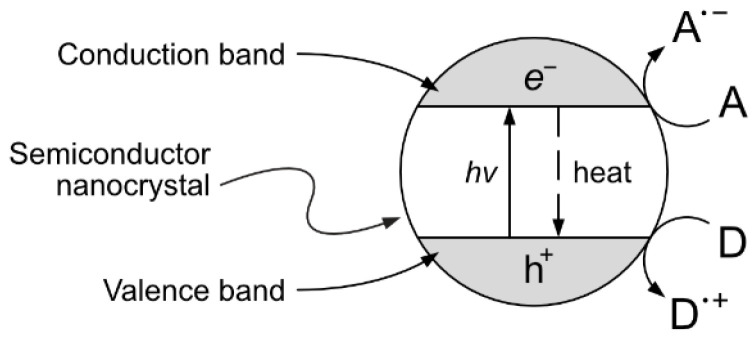
Heterogeneous photocatalytic process. The adsorption of a photon on a semiconductor crystal causes the promotion of an electron from the valence band to the conduction band, generating an e^−^/h^+^ pair in the bulk of the photocatalyst. The generated electron and hole can then migrate to the surface and become available for redox reactions with an electron acceptor (A) or donor (D) species or they can thermally recombine in a competitive process.

One interesting approach, although still at the experimental stage, is the production of vanillin via controlled oxidation of lignin using photocatalytic techniques. The implementation of this strategy is particularly appealing because of the potential sustainability of the resulting process. The selection of studies given in the following paragraphs can provide a good starting point for the development of further research. Heterogeneous photocatalysis is based on the photon-driven promotion of an electron from the valence band to the conduction band, resulting in a catalytic activated semiconductor crystal. The formed positive hole (h^+^) and free electron (e^−^) can then migrate from the bulk to the surface of the catalyst crystal, becoming available for redox reactions with suitable electron donors and acceptors (see Figure 13).

The possible (and usually favored) electron–hole recombination in the catalyst structure is, however, a direct concurrent result of the desired surface redox processes. Therefore, great research efforts have been devoted to ease the redox reaction (e.g., by reducing the photocatalyst crystal size in order to reduce the length of the charge migration path from the bulk to the surface) and/or stabilize the generated e^−^/h^+^ pair enhancing the charge separation (e.g., by synthesizing composite photocatalysts with tailored band structures that favor the physical displacement of electrons and holes) [103]. A substantial role in water-based heterogeneous photocatalytic oxidation processes can be also given by the hydroxyl radical HO·, which can be generated from the direct oxidation of water at the catalyst surface and other secondary processes [104].

Because of the photon contribution to the reach of the reaction activation energy, photocatalytic reactions can be easily carried out in mild conditions at ambient temperature, typically using water as a solvent and without resorting to toxic oxidizing or reducing agents. Photocatalytic processes are thus considered a very interesting option in the context of green chemistry. Moreover, photocatalytic reactions can be potentially carried out directly exploiting natural solar radiation as a photon source, further reducing the environmental impact of the implemented process [105]. Several applications of photocatalysis have been studied, including water splitting for hydrogen production [106], carbon dioxide reduction [107] and implementations such as advanced oxidation processes (AOPs) for air [108] or water [109] depollution and sustainable chemical process development [110,111].

Not surprisingly, photocatalytic technologies quickly received a notable interest also for the development of lignin oxidation processes [112], with an initial focus on environmental applications such as wastewater depollution [113] and then, in the last few years, on the controlled lignin depolymerization for the sustainable production of chemical intermediates [114,115]. The controlled oxidation of lignin to obtain vanillin or other chemical derivatives is a multi-step process, with several intermediates and different possible pathways involved. Competitive degradation routes resulting in different products and parallel degradation processes of the formed vanillin are both common causes of reduced yields. A possible transient intermediate in the oxidative synthesis of vanillin from lignins is isoeugenol [116]. The direct synthesis of vanillin via the heterogeneous photocatalytic oxidation of eugenol, isoeugenol, trans-ferulic acid and vanillic alcohol was studied by Agugliaro et al. [117] in aqueous medium. Common parasitic in-process degradation routes of the formed vanillin are the oxidation to vanillic acid and the formation of dihydroxybenzene derivatives due to a hydroxyl radical attack to the aromatic ring (typically followed by further attacks to give short-chain organic acids via ring openings [116]). Dimerization and repolymerization are also of concern in these conditions [118,119].

#### 3.5.1. Photocatalysis of Pulping Black Liquor

Prado et al. [120] reported an example of photocatalytic lignin depolymerization directly performed on the pulping black liquor (i.e., without lignin separation) for the recovery of derived compounds. The authors used two different black liquors deriving respectively from organosolv pulping (with 60% ethanol at 180 °C for 90 min, solid/liquid ratio 1:4) and from ionic liquid (IL) pulping (with [Bmim][MeSO_4_] under microwave, 200 °C for 30 min, solid/liquid ratio 1:10). The photocatalytic oxidation was carried out adding the photocatalyst (sol–gel synthesized TiO_2_) to the pulping black liquor (2 g/L for organosolv-derived black liquor and 4 g/L for the IL-derived one). After photocatalysis, lignin was separated via acid precipitation and centrifugation while the liquid fraction was extracted with ethyl acetate to obtain the derived oil. The analysis of the precipitated lignin demonstrated a better degradation from the photocatalysis of organosolv black liquor in comparison to the photocatalyzed IL black liquor. This was attributed to the different pH values of the two reaction media (4.8 for organosolv vs. 7.0 for IL). In the extracted oil, the authors observed lignin-derived compounds and also furfural and other sugar degradation products. The yields of lignin-derived phenolics were definitely higher for the organosolv pulping medium. One of the main recovered compounds in the extracted oil was syringaldehyde, which accounted for up to 14.2% (*w*/*w*) of the oil fraction for organosolv black liquor and 1.2% for IL black liquor (both obtained after 0.5 h photocatalysis). The maximum recovered vanillin was 0.9% (organosolv) and 0.1% (IL), always as the oil fraction extracted after 0.5 h of photocatalytic treatment. Longer photocatalysis reaction times resulted in lower yields. The remarkably lower yields from IL black liquor photocatalysis were attributed to the formation of nitrogen-containing compounds derived from the photocatalytic degradation of the ionic liquid components. Although this is interesting from the point of view of process economy (no lignin separation must be performed), the reported results indicate that the direct photocatalytic treatment of pulping black liquors could be critical in most aspects, including the sub-optimal condition dictated by the pulping medium itself (e.g., pH) and the presence of critical amounts of extraneous compounds (e.g., sugars and pulping reagents) that can interfere with the photocatalytic process, both lowering the yields and generating undesirable byproducts.

#### 3.5.2. Photocatalysis of Separated Lignins

The problems encountered in the direct photocatalysis of black liquor can be overcome by operating on lignins purified from the pulping medium. The application of photocatalysis to the synthesis of vanillin from sodium lignosulfonate (SLS) has been recently reported by Qiu et al. [121]. The authors used a mesoporous, high specific surface area titanium dioxide photocatalyst obtained from the calcination in air of MIL-125, a Ti-based metal–organic framework. The photocatalytic conversion experiments were carried out with Xenon lamp irradiation (6 h) under air at room temperature. Of the three different catalysts prepared, calcinated at 400, 500 and 600 °C, respectively, the one calcinated at 400 °C demonstrated the highest specific surface area (174 m^2^/g) and the highest vanillin yields (2.1 mg per g of SLS). As a reference, the same process carried out with a standard titania photocatalyst (Degussa P25, 48 m^2^/g specific surface area) result in an order of magnitude minor yield (0.27 mg per g of SLS). Interestingly, conversion experiments conducted in the presence of specific trapping agents (isopropanol, EDTA and p-benzoquinone for HO·, h^+^ and O_2_·^ −^, respectively) indicate the active role of h^+^ and O_2_· ^−^ (the suppression of which causes a marked decrease in vanillin yields). The suppression of HO· causes instead a rise in vanillin yield (that rises to 2.9 mg per g of SLS), indicating the negative role of hydroxyl radicals in the process, possibly due to the activation of both concurrent oxidation pathways on the lignosulfonate backbone and degradative oxidation of the produced vanillin.

Ahmad et al. [122] described the formation of vanillin and 4-hydroxybenzaldehyde in the photocatalytic degradation of lignin obtained via the delignification of rice straw residues. The lignin extraction was performed in 1 M NaOH solution at 150 °C for 1 h. The photocatalytic alkaline lignin extraction was performed in a stirred reactor using TiO_2_ or ZnO-suspended photocatalyst particles. While the lignin underwent a steady degradation during the photocatalytic process, the concentration of both vanillin and 4-hydroxybenzaldehyde demonstrated, after an initial induction phase where none of the two products were detected, a rise and a following decrease after they reached the maximum value. This was attributed to the activation of the concomitant photocatalytic degradation of both products, confirmed by the authors who measured the degradation kinetics of pure vanillin and 4-hydroxybenzaldehyde in separate experiments using the same photocatalytic conditions. The maximum lignin degradation rate was found for ZnO with 2 g/L catalyst loading and, in these conditions, a higher vanillin concentration was also recorded (51.2 mg/L at 8 h process time). The 4-hydroxybenzaldehyde was formed in lower amounts, with the maximum concentration (20.4 mg/L) recorded using the TiO_2_ photocatalyst at 1.5 g/L catalyst loading at 10 h process time.

Tonucci et al. [123] studied the photocatalytic degradation of ammonium (AmLig) and calcium (CaLig) lignosulfonates in very mild conditions comparing the performances of a well-known heterogeneous photocatalyst (TiO_2_, Degussa P25) with three polyoxometalates (POMs) as homogeneous photocatalysts (POM-1: H_5_[PMo_10_V_2_O_4_]·H_2_O; POM-2: K_5_[Ru(H_2_O)PW_11_O_39_]; POM-3: K_4_[SiW_12_O_40_]·8H_2_O). The three POMs were also tested as thermal catalysts (i.e., without irradiation), and a further experiment was carried out using the Fenton reagent (FeSO_4_/H_2_O_2_). All experiments lasted for maximum of 24 h in aqueous lignin solution (2.5% *w*/*w*) at ambient temperature. The Fenton reagent demonstrated a high degradation of both lignins (residual levels < 30%, mainly constituted by short chain mono and dicarboxylic acids), so it was considered not appropriate for the efficient recovery of monomeric derivatives. Interestingly, when used in homogeneous photocatalytic conditions, the three POMs demonstrated the best vanillin recovery performance of all the studied systems, with particular reference to POM-2 (71 and 133 mg/L of vanillin from AmLig and CaLig, respectively) and POM-3 (105 and 125 mg/L of vanillin from AmLig and CaLig). This compared with the quite low recovery obtained with TiO_2_ (15 and 6 mg/L of vanillin from AmLig and CaLig, and these values were essentially comparable with the recovery from blank solutions). However, the authors found remarkably low stability of the three POMs under photocatalytic conditions, which makes the prospect of their effective use potentially problematic.

A common way for enhancing photocatalytic activity is the synthesis of a composite photocatalysis constituted by two different phases (e.g., semiconductor/semiconductor or metal/semiconductor) in intimate contact in order to establish a junction that allows for the free exchange of electrons and holes. The different energy levels of the charges in the two phases drive the selective migration of electrons and holes promoting a physical charge separation that reduces recombination, ultimately increasing their availability for redox reactions at the surface. An example of a metal/semiconductor composite photocatalyst (Pt/TiO_2_) applied to lignin oxidation was proposed by Ma et al. [124] in the context of complete degradation for wastewater treatment.

A similar but more product-oriented approach was proposed by Gong et al. [125] in the selective photocatalytic oxidation of sodium lignosulfonate using several platinum and bismuth co-modified TiO_2_ catalysts. The lignin photocatalytic oxidation activity was determined in water solutions at different pH values, using a 1 g/L catalyst concentration and 100 mg/L lignin concentration. The authors found the highest lignin conversion (84.5%) for the Bi_0.01_/Pt_0.01_-TiO_2_ catalyst after 1 h of irradiation. The total byproducts yield was 23.2%, with different identified products including, among others, guaiacol, vanillic acid and vanillin. In this study, however, vanillin was qualitatively detected but not quantified, and therefore specific yields were not available. The charge transfer from TiO_2_ to Pt and Pt/Bi was considered the main enhancing factor for the photocatalytic activity. The initial pH results had little effect on the lignin degradation yields but a remarkable impact on the reaction selectivity. Moreover, the product selectivity was affected also by the relative amount of Bi and Pt in the catalyst. Interestingly, quenching experiments (carried out by the authors with acid orange as the target compound instead of lignin to avoid unwanted interferences) indicated also that, in the presence of Bi, the main active species were h^+^ and O_2_· ^−^ with a reduced importance of hydroxyl radicals. The Pt appeared to enhance this behavior, with an even more reduced role of HO· that can help to explain the higher selectivity. These results indicated the importance of controlling the effective reaction pathways in order to enhance the selectivity for the desired products.

Du et al. [126] studied the use of a fullerene (C_60_)-modified Bi_2_TiO_4_F_2_ photocatalyst in the controlled oxidation of Kraft lignin. Fullerene is an allotropic form of carbon composed of 60 sp^2^ hybridized atoms organized in a closed cage structure. Irradiated fullerenes can reach a long-life (>40 µs) excited triplet state (^3^C_60_*) that is a good electron acceptor and, upon interaction with a suitable donor, can be reduced to the singlet radical anion (^1^C_60_·^−^). In composite fullerene-semiconductor photocatalysts, the *e^−^* promoted to the semiconductor conduction band can thus migrate to the fullerene structure, establishing a good electron–hole separation that inhibits the recombination and enhances the photocatalytic activity [127]. The authors synthesized the composite C_60_/Bi_2_TiO_4_F_2_ photocatalysts and, as a reference, the composite C_60_/TiO_2_ and the pure Bi_2_TiO_4_F_2_. The photocatalytic lignin degradation activity of the three synthesized catalysts was studied under visible light (500 W xenon lamp with a UV cut-off filter) in 12 h process runs at room temperature under air bubbling to ensure a proper oxygen level. The authors found a strong pH-dependent activity that could be attributed both to the decreased light transmittance of the lignin solution at higher pH values and to the effect of pH on the lignin adsorption on the catalyst surface. The optimal found values were pH 7 for the two composite catalysts and pH 8 for Bi_2_TiO_4_F_2_. The found catalyst efficiency was in the order C_60_/Bi_2_TiO_4_F_2_ > Bi_2_TiO_4_F_2_ > C_60_/TiO_2_, which is consistent with the UV-Vis absorption spectra of the three photocatalysts. The authors found the same main lignin degradation products for all the studied photocatalysts but in different yields. Particularly, for C_60_/Bi_2_TiO_4_F_2_, they observed homovanillic acid as the main product followed by vanillin and 2-methoxyphenol. The recorded yields for the same catalyst were >2.5% for homovanillic acid, >0.5% for vanillin and >0.2% for 2-methoxyphenol at 1.25 g/L lignin concentration. The relative product yields and the total relative conversion decreased at higher lignin concentrations but not very sharply, so the maximum total yields were observed for all catalysts at 5~6 mg/L concentration level. The authors studied also the stability of the three photocatalysts with a five-cycles reuse experiment, finding a >50% relative activity decrease for Bi_2_TiO_4_F_2_ and about half of this value for C_60_/Bi_2_TiO_4_F_2_ and C_60_/TiO_2_ (25.5% and 28.3%, respectively). Interestingly, energy-dispersive X-ray spectroscopy demonstrated that the Bi content was reduced in both the recycled Bi_2_TiO_4_F_2_ and C_60_/Bi_2_TiO_4_F_2_ but in a lesser extent for the latter, suggesting that the interaction with C_60_ can reduce the loss of Bi thus containing the deactivation rate.

#### 3.5.3. Photocatalysis with Combined Processes

A possible strategy for enhancing the overall performance of photocatalytic oxidation is the combination with other complementary approaches developing an integrated process with optimized performances. Few works involving the specific production of vanillin are available in the literature on this topic. An example targeted at lignin oxidation was proposed by Tian et al. [128] which described a combination of photocatalytic and electrochemical processes for the degradation of Kraft lignin. The authors studied various combinations of a Ti/TiO_2_ nanotube electrode and a Ti/TaO_5_-IrO_2_ electrode in electrochemical and/or photocatalytic operation modes. The counter electrode was a Pt foil, while an Ag/AgCl electrode was used as a reference electrode. The authors studied the kinetics of the lignin degradation process measuring the lignin concentration with UV spectrophotometry (295 nm). All studied processes resulted in pseudo-first-order kinetics. The highest rate constant (0.021 min^−1^) was demonstrated by the combined photocatalytic/electrochemical process obtained and by connecting both electrodes at +600 mV (vs. the Ag/AgCl reference electrode) under UV irradiation. The separate electrochemical oxidation, performed with the Ti/TaO_5_-IrO_2_ electrode held at +600 mV without UV irradiation resulted in less than half the degradation rate constant (0.009 min^−1^). Interestingly, the separate photocatalytic process performed under UV irradiation with an unpolarized Ti/TiO_2_ electrode gave the lowest rate constant (0.0025 min^−1^), while the same experiment repeated with +600 mV polarization of the electrode resulted in a doubled rate value (0.005 min^−1^), indicating an enhanced efficiency of the process attributed to the suppression of the electron/hole recombination due to the applied potential bias. Overall, these results indicated that the combined photocatalytic–electrochemical process was more efficient than the sum of the two processes alone, suggesting a synergetic effect in lignin oxidation. The presence of vanillin and vanillic acid in the residue derived from a 12 h combined process run was qualitatively confirmed via HPLC and GC-MS, but no quantitative determinations were performed.

The combined photocatalytic and biocatalytic degradation of Kraft lignin was studied by Kamwilaisak et al. [116] using TiO_2_ and laccase. The authors studied both a combined dual-step process (photocatalysis followed by biocatalysis) and a combined single-step process (photocatalysis and biocatalysis running concurrently in the same batch). Separate photocatalysis and biocatalysis processes were performed as references. All batches were carried out at 50 °C and pH 5 for 24 h using 1 g/L lignin and 3 g/L TiO_2_ (for photocatalytic processes) and 2.5 units/mL laccase (for biocatalytic processes). The photocatalytic processes were carried out under UV-A light by fluorescent tubes at 5.3 µE cm^−2^ s^−1^ (~1.7 W/cm^2^ at 370 nm). The authors studied also the effect of the addition of H_2_O_2_ (5.55 g/L) in all experiments. The lignin degradation yield of the different systems was biocatalysis << photocatalysis ≈ combined single-step < combined dual-step. The addition of H_2_O_2_ increased the degradation yield of all the processes with the sole exception of the combined single-step. Several degradation products were identified, mainly, organic acids and carbohydrates. Vanillin was identified but not quantified. This study indicated the potentialities of combined strategies but also the possible problems due to the interference of different processes running simultaneously in the same reactor.

A completely different approach was proposed by Miyata et al. [129] with an original solid-state photocatalytic delignification process of wood powders. The authors observed that in the radical autooxidation of polyolefins, the rate-determining step was the chain branching following the decomposition of the neo-formed hydroperoxide and that this reaction was analogous to the Cα–Cβ bond cleavage reaction in delignification. Moreover, the same authors demonstrated, in a previous work, that in performing a photocatalytic oxidation of polypropylene with TiO_2_ in the presence of poly(ethylene) oxide (PEO), a significant amount of acids and aldehydes was produced, and this facilitated the hydroperoxide decomposition, greatly enhancing the polymer degradation. Consequently, a process based on TiO_2_/PEO was proposed for the solid-state delignification of previously defatted wood flour samples. The wood powder (particle size <1 mm) was defatted via two 48 h runs in a Soxhlet apparatus with acetone and benzene/ethanol (2:1 *v*/*v*), respectively. The photocatalyst was applied to the treated wood powder (10 g) as an aqueous solution (56 g) containing TiO_2_ (33 mg) and PEO (527 mg). The sample was exsiccated and then put on a Petri dish for UV exposure (400 W mercury vapor lamp at 50 cm, 48 h, 30 °C). While the delignification was apparent in a SEM analysis of the processed powder, the effective delignification yield was quite low (from 28.22% to 27.96%, gravimetric). This result was attributed also to the difficult spreading of the radical species generated by TiO_2_/PEO in the inner region of the wood particles. The authors, however, qualitatively demonstrated (with ^1^H-NMR) the presence of vanillin in the soluble extraction fraction of the treated sample.

While all the described research are still pioneering works, they show the high potential of the integrated process approach, and more research is definitely needed on this topic to fully exploit all the possible synergies given by the combination of complementary technologies.

#### 3.5.4. Perspectives for Vanillin Production by Controlled Photocatalytic Lignin Degradation

Although currently still at the experimental laboratory stage, the production of vanillin by the controlled photocatalytic oxidation of lignin appears to be a potentially very interesting goal, particularly considering the increasing focus on the sustainability of industrial chemical processes. However, several problems must still be resolved, and more research is needed for an effective deployment of the technology. Despite the overall mild operating conditions, the typically high amount of hydroxyl radicals generated in the photocatalytic process can establish a quite reactive environment that possibly causes a significant degradation of the generated vanillin. Catalytic systems with enhanced selectivity are needed for an effective production of the target molecule, reducing competitive degradation pathways of the original lignin and of the product itself. In this regard, it is interesting to note that the mild operating conditions potentially enable the use of tailored on-process separating systems such as membranes or adsorbing resins in order to separate the neo-formed vanillin from the reaction medium, thus avoiding possible product degradation. Original approaches on the reactor construction may therefore be interesting research topics [130]. Moreover, catalyst deactivation can always be an issue, especially in the case of complex catalytic systems. The development of mechanically anchored catalytic systems can also address the problems related to the recovery of heterogeneous catalysts dispersed as powder in the process medium. All these considerations apply also to other low molecular weight products that can be potentially obtained from lignin.

## 4. Conclusions and Challenges

Vanillin has been known, for a long time, for its properties as a flavoring agent for food, beverages and pharmaceuticals. Moreover, its belonging to the large class of phenolic compounds has pushed the research more recently on its antioxidant properties and its use as a natural building block for many biobased polymers. In this framework, vanillin obtained from a biobased waste residue such as lignin offers various significant environmental and economic benefits including waste reduction and natural resource recycling. The development of new technologies and processes for the production of vanillin from lignin thus represents a promising approach to valorize an underutilized renewable resource and also address the growing global demand for natural vanillin. Chemical, enzymatic, microbial and photochemical conversion methods have been described here and have shown great potentialities in providing a more sustainable and cost-effective source of vanillin. However, it appears that highly significant challenges remain in terms of vanillin yield, selectivity, and process efficiency.

The main point that has emerged in the present review is that, even if great successes in the research have been obtained in the last few years, the efficient conversion of lignin to vanillin is still hindered by the complex and heterogeneous nature of lignin, the limited selectivity of conversion and the very low yields of the processes. Not surprisingly, among the various strategies examined, those with the greatest energy and environmental promise are also those that present the greatest difficulties and require the most intense research. For these reasons, the presented methodologies are mostly on a lab-scale level, and the majority of the commercial vanillin is still not natural. However, the approaches reported here represent an important backbone to plan new strategies to produce marketable vanillin from lignin. In order to develop innovative approaches for obtaining vanillin from lignin with sustainable processes, the following research topics therefore appear particularly interesting:

1. The development of tailored lignin extraction methods to obtain lignin fractions optimized for vanillin production;
2. The study of combined strategies (such as enzymatic/microbial, photocatalytic/electrochemical and photocatalytic/enzymatic processes) in order to exploit the advantages of different approaches and enhance yields and efficiency;
3. The development of efficient separation techniques for vanillin extraction and purification, either as a downstream separate step or as a simultaneous process (e.g., vanillin adsorption by resins during biocatalytic or electrochemical batch conversion);
4. The investigation of novel catalysts and enzymes that exhibit high selectivity and activity toward vanillin production;
5. The exploitation of advanced metabolic engineering and synthetic biology with the help of computational modelling approaches to build microbial strains with optimized vanillin biosynthesis pathways;
6. The development of life cycle assessment models for lignin valorization from an economic, technological and environmental perspective.

From an industrial point of view, the main strategy to valorize lignin for the production of monomers of pharmaceutical, cosmetic and flavor interest and of polymers, lies in its inclusion in biorefineries. In fact, usually, only cellulose and hemicellulose are used as platforms in biorefineries, while lignin is considered a waste, because of its heterogeneity and complexity. However, in the future, a “lignin first” approach should be embraced, and lignin valorization should be the main part of the biorefineries, allowing us to reduce the main issues of the current processes. Nevertheless, this approach is still at the beginning, since different conversion and purification methodologies need to be tested at a higher scale according to the desired product, but this will allow us to improve the economic and environmental aspects of the overall processes. Finally, a new business model on the use of lignin needs to be developed in order to shed light on the real contribution of lignin valorization to circular economy.

It is worth also to note that these lines of research, as well as the various considerations given in the present paper, are not specific only for vanillin production but could be replicable to the more general (and very present) problem of obtaining valuable aromatic intermediates from a low-cost waste product such as lignin. Any specific activity in this area therefore has an inherent potential for reapplication across the entire field of lignin valorization, allowing for a broad synergy of research activities and an enhanced possibility of return on investment.
